# Truncated Class 1 Integron Gene Cassette Arrays Contribute to Antimicrobial Resistance of Diarrheagenic *Escherichia coli*

**DOI:** 10.1155/2020/4908189

**Published:** 2020-01-31

**Authors:** Akiko Kubomura, Tsuyoshi Sekizuka, Daisuke Onozuka, Koichi Murakami, Hirokazu Kimura, Masahiro Sakaguchi, Kazunori Oishi, Shinichiro Hirai, Makoto Kuroda, Nobuhiko Okabe

**Affiliations:** ^1^Kawasaki City Institute for Public Health, 3-25-13 Tonomachi, Kawasaki-ku, Kawasaki, Kanagawa 210-0821, Japan; ^2^Pathogen Genomics Center, National Institute of Infectious Diseases, 1-23-1 Toyama, Shinjuku, Tokyo 162-8640, Japan; ^3^Department of Preventive Medicine and Epidemiology, National Cerebral and Cardiovascular Center, 6-1 Kishibe-Shimmachi, Suita, Osaka 564-8565, Japan; ^4^Infectious Disease Surveillance Center, National Institute of Infectious Diseases, 4-7-1 Gakuen, Musashi-Murayama, Tokyo 208-0011, Japan; ^5^School of Medical Technology, Faculty of Health Sciences, Gunma Paz University, 1-7-1 Tonyamachi, Takasaki-shi, Gunma 370-0006, Japan; ^6^Department of Veterinary Microbiology I, School of Veterinary Medicine, Azabu University, 1-17-71 Fuchinobe, Chuo-ku, Sagamihara, Kanagawa 252-5201, Japan; ^7^Toyama Institute of Health, 17-1 Nakataikoyama, Imizu, Toyama 939-0363, Japan

## Abstract

Class 1 integrons (c1-integrons) are associated with multidrug resistance in diarrheagenic *Escherichia coli* (DEC). However, little is known about gene cassettes located within these c1-integrons, particularly truncated c1-integrons, in DEC strains. Therefore, the aims of the present study were to reveal the relationship between antimicrobial resistance and the presence of truncated c1-integrons in DEC isolates derived from human stool samples in Japan. A total of 162 human stool-derived DEC isolates from Japan were examined by antimicrobial susceptibility testing, PCR-based gene detection, and next-generation sequencing analyses. Results showed that 44.4% (12/27) of c1-integrons identified in the DEC isolates harbored only *intI*1 (an element of c1-integrons) and were truncated by IS*26*, Tn*3*, or IS*1*-group insertion sequences. No difference in the frequency of antimicrobial resistance was recorded between intact and truncated c1-integron-positive DEC isolates. Isolates containing intact/truncated c1-integrons, particularly enteroaggregative *E. coli* isolates, were resistant to a greater number of antimicrobials than isolates without c1-integrons. *aadA* and *dfrA* were the most prevalent antimicrobial resistance genes in the intact/truncated c1-integrons examined in this study. Therefore, gene cassettes located within these intact/truncated c1-integrons may only play a limited role in conferring antimicrobial resistance among DEC. However, DEC harboring truncated c1-integrons may be resistant to a greater number of antimicrobials than c1-integron-negative DEC, similar to strains harboring intact c1-integrons.

## 1. Introduction

**Figure 1:**
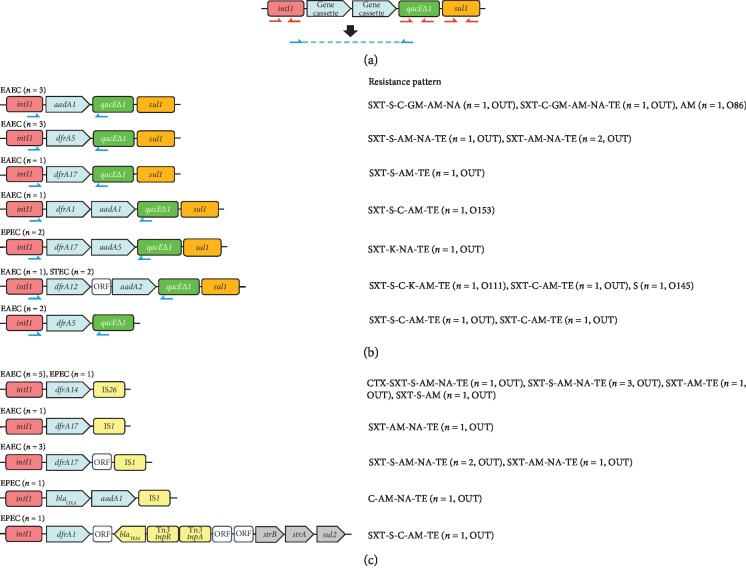
(a) General structure of class 1 integrons (c1-integrons). The red arrows show the positions of primers used for detection of *intI1*, *qacEΔ1*, and *sul1*. The blue arrows show the positions of primers used for sequencing. P indicates the promoter. (b) Intact c1-integron cassette arrays that were confirmed by sequencing of PCR products, along with the corresponding resistance patterns. (c) Truncated c1-integron cassette arrays that were confirmed by next-generation sequencing analysis, along with the corresponding resistance patterns. EAEC: enteroaggregative *Escherichia coli*; EPEC: enteropathogenic *E. coli*; STEC: Shiga toxin-producing *E. coli*; CTX: cefotaxime; SXT: sulfamethoxazole-trimethoprim; (S) streptomycin; (C) chloramphenicol; (K) kanamycin; AM: ampicillin; NA: nalidixic acid; TE: tetracycline; *aadA1*: aminoglycoside resistance gene; *dfrA*: dihydrofolate reductase gene (trimethoprim resistance); OUT: O-serogroup-untypeable. All insertion sequences designated “IS*1*” belong to the IS*1* family.

Gene cassettes located within class 1 integrons (c1-integrons) may play an important role in diarrheagenic *Escherichia coli* (DEC) strains. DEC are generally classified into five categories (enterotoxigenic *E. coli* (ETEC), enteropathogenic *E. coli* (EPEC), Shiga toxin-producing *E. coli* (STEC), enteroaggregative *E. coli* (EAEC), and enteroinvasive *E. coli*) on the basis of their virulence traits [1]. Among the categories, EPEC and EAEC are known for their high prevalence in both community and/or clinical settings [2, 3]. EAEC strains display higher rates of resistance to several antibiotics when compared with that of other DEC pathotypes [4, 5]. c1-integrons are a major source of antibiotic resistance genes and contain three main elements: an integrase gene (*intI*), a primary recombination site (*attI*), and a strong promoter. c1-integrons capture gene cassettes conferring resistance to antibiotics via IntI-catalyzed recombination between the *attI* recombination site and a 59-bp element called *attC* present on the gene cassettes (Figure 1(a)) [6]. c1-integron-harboring bacterial strains generally show higher rates of antibiotic resistance than those without c1-integrons [7, 8]. Moreover, the presence of c1-integrons contributes to multidrug resistance (MDR), defined as resistance to three or more classes of antimicrobials, in Enterobacteriaceae [9]. While little is known about gene cassettes located within intact c1-integrons in DEC strains [6], even less is known about genes found in truncated c1-integrons. Therefore, further research is needed to evaluate gene cassettes in truncated c1-integrons.

There is also a lack of information about the role of truncated c1-integrons in the antimicrobial resistance of DEC. Previous work has shown that truncated c1-integrons are involved in the dissemination of antimicrobial resistance genes such as *bla*_SHV-12_ and *bla*_VIM-7_ in bacteria other than DEC, including *Enterobacter cloacae* [10] and *Pseudomonas aeruginosa* [11], respectively. Thus, truncated c1-integron cassettes should be evaluated in DEC. However, it is difficult to investigate gene arrays in truncated c1-integron cassettes because repeat sequences, insertion sequences (IS), and transposons can result in truncation of the genes, inhibiting amplification reactions [12]. As such, the aims of the present study were to reveal the relationship between antimicrobial resistance and the presence of truncated c1-integrons in DEC isolates derived from human stool samples in Japan using both conventional sequencing and next-generation sequencing (NGS) analyses.

## 2. Materials and Methods

### 2.1. Bacterial Strains

A total of 162 DEC isolates, consisting of 40 EAEC, 37 EPEC, 83 STEC, and two ETEC, were examined. All DEC isolates, except for 51 of the STEC isolates, were obtained from stool samples collected from asymptomatic carriers and patients with gastrointestinal symptoms at the Kawasaki City Institute for Public Health, Japan, from 2012 to 2014. The remaining 51 STEC isolates were collected from outpatients at several hospitals in Kawasaki between April 2012 and December 2014 (Table 1). The 40 EAEC isolates were also examined in our previous study of antimicrobial resistance patterns [13]. The ETEC and EPEC isolates were identified by PCR-based assays using primers targeting *eae* [14] and *elt* and *est* (primers ELT−1/−2, ESH−1/−2, and ESP−1/−2; Takara Biomedicals, Kusatsu, Japan). The 83 STEC isolates were identified using *stx*-targeting PCR primers EVC−1/−2 (Takara Biomedicals) or using a Loopamp Verotoxin Typing Kit (Eiken Chemical Co., Tokyo, Japan). For all of the DEC isolates, O-serotyping was conducted using a slide agglutination method with 43 commercially available O-antisera (Denka Seiken Co., Tokyo, Japan).

### 2.2. Antimicrobial Susceptibility Testing

Antimicrobial susceptibility profiles were determined using the disc diffusion method with BD Sensi-Discs (Becton Dickinson, Tokyo, Japan) according to the guidelines outlined in Clinical and Laboratory Standards Institute documents M02-A13 and M100-S28 [15, 16]. The following 14 antibiotic discs were used: cefotaxime (30 *μ*g), norfloxacin (10 *μ*g), sulfamethoxazole-trimethoprim (23.75 *μ*g, 1.25 *μ*g), streptomycin (10 *μ*g), chloramphenicol (30 *μ*g), ciprofloxacin (5 *μ*g), kanamycin (30 *μ*g), gentamicin (10 *μ*g), ampicillin (10 *μ*g), fosfomycin (50 *μ*g), nalidixic acid (30 *μ*g), tetracycline (30 *μ*g), imipenem (10 *μ*g), and meropenem (10 *μ*g).

### 2.3. Detection of c1-Integrons

DNA template was extracted from each isolate using a QIAamp DNA Stool Mini Kit (Qiagen GmbH, Hilden, Germany). In general, the 5ʹ-conserved segment (5ʹCS) of c1-integrons contains *intI1*, while the 3ʹ-conserved segment (3ʹ-CS) contains both *qacEΔ1* and *sul1* (Figure 1(a)). In this study, the presence of a c1-integron was confirmed by three independent amplifications of *intI1*, *qacEΔ1*, and *sulI* (Figure 1(a)) via PCR-based assays [17].

### 2.4. Amplification and Sequencing of Gene Cassette Regions

The isolates from which genes in the 5ʹ-CS and 3ʹ-CS regions could be amplified were classed as containing intact c1-integrons. These isolates were then subjected to PCR using the 5ʹCS/3ʹ-CS primers, followed by Sanger sequencing of the resulting amplicons to determine the sequence of the region between *intI1* and *qacEΔ1* in intact c1-integrons (Figure 1(a)) [17]. Acquired resistance genes within each c1-integron were analyzed using the ResFinder platform (http://genomicepidemiology.org/), while similarity searches were performed using BLAST (http://www.ncbi.nlm.nih.gov/BLAST/) [18, 19]. The primers used for PCR analyses are described in Table 2.

### 2.5. Next-Generation Sequencing

Integrons from which only *intI1* could be amplified (i.e., missing *qacEΔ1* and *sulI*) were classed as truncated integrons. Isolates harboring truncated c1-integrons were subjected to next-generation sequencing analysis. DNA extraction from the strains was carried out as described previously [20]. A short insert size (approximately 0.5 kb) paired-end library was constructed using a Nextera XT DNA Library Prep Kit (Illumina, San Diego, CA, USA), followed by whole-genome sequencing using the Illumina NextSeq 500 platform with a 300-cycle NextSeq 500 Reagent Kit v2 (2 × 150 mer). The extracted contigs were validated by comparison against the whole-genome sequence database GenEpid-J [21] and by using the ResFinder and VirulenceFinder tools available from the Center for Genomic Epidemiology (http://www.genomicepidemiology.org/).

### 2.6. Detection of Extended-Spectrum *β*-Lactamase (ESBL) Genes

Isolates that showed resistance to cefotaxime during antimicrobial susceptibility testing were further examined for the presence of ESBL genes (*bla*_CTX-M_, *bla*_TEM_, and *bla*_SHV_) by PCR, as described previously [22, 23]. Primers used for sequencing of *bla*_TEM_ were designed in this study: TEMseq-F, 5ʹ-GGTGCGGTATTATCCCGTGT-3ʹ; TEMseq-R, 5ʹ-TTGTTGCCGGGAAGCTAGAG-3ʹ. The resulting PCR amplicons were sequenced and the nucleotide and deduced amino acid sequences were compared with entries in the GenBank database (http://www.ncbi.nlm.nih.gov/BLAST/), as well as with those described on the *β*-lactamase classification website (http://www.lahey.org/Studies/, accessed February 2017), to determine the *β*-lactamase gene subtype.

### 2.7. Statistical Analyses

Statistical analyses were performed using Fisher's exact test. A *p*-value of <0.05 was considered statistically significant.

### 2.8. Ethical Approval

This study was performed in accordance with the guidelines of the Ethics Regulations Related to Medical Research Involving Human Subjects at the Kawasaki City Institute for Public Health under permit number 28-2.

## 3. Results

### 3.1. Susceptibility to Antimicrobial Agents

Of the 162 DEC isolates, 64 were resistant to at least one of the antibiotics tested, with 32 isolates showing MDR (Table 3). As shown in Table 4, the highest prevalence of antimicrobial resistance was associated with ampicillin (50 isolates, 30.9%), followed by tetracycline (39 isolates, 24.1%) and sulfamethoxazole-trimethoprim (28 isolates, 17.3%). Furthermore, EAEC isolates showed resistance to a greater number of antimicrobial agents than EPEC or STEC isolates. MDR phenotypes were more frequently associated with DEC (*p* < 0.001), EAEC (*p* < 0.0011), and EPEC isolates (*p*=0.002) harboring intact/truncated c1-integrons than with c1-integron-negative isolates (Table 4).

### 3.2. Frequency of c1-Integrons

EAEC isolates were more likely to harbor intact/truncated c1-integrons (50.0%) compared with EPEC (13.5%), STEC (2.4%), and ETEC (0%) isolates (Tables 3 and 4). Within DEC pathotypes EAEC, EPEC, and STEC isolates containing c1-integrons had significantly higher rates of resistance to seven specific drugs compared with c1-integron-negative isolates (Tables 3 and 4).

### 3.3. Identification of Gene Cassette Arrays within c1-Integrons

NGS analysis revealed that the 3ʹ-CS regions of the 12 isolates harboring truncated c1-integrons were truncated by the insertion of IS*26* (*n* = 6), insertion sequences belonging to the IS*1* group (*n* = 5), or by transposon Tn*3* (*n* = 1) (Figures 1(b) and 1(c)). No differences in the frequency of antibiotic resistance or in the number of antibiotics to which isolates showed resistance were observed between isolates harboring intact and truncated c1-integrons (Table 3).

Overall, 7.5% (3/40) of EAEC isolates and 2.7% (1/37) of EPEC isolates contained ESBL genes, none of which were identified in the STEC or ETEC isolates. Importantly, none of the ESBL genes detected in the current study (three *bla*_CTX-M-14_ and one *bla*_CTX-M-15_; Table 4) were located within the c1-integron cassettes.

### 3.4. Nucleotide Sequences

All sequence data for c1-integrons amplified using primers 5ʹCS/3ʹ-CS have been deposited in the GenBank database under accession numbers LC380541–LC380554 and LC383355. Raw sequence reads have been deposited in the DNA Data Bank of Japan Sequence Read Archive under Biosample IDs SAMD00117734–SAMD00117745 (Run IDs DRR129827–DRR129838) (Supplementary [Supplementary-material supplementary-material-1]).

## 4. Discussion

Based on the results of this study, DEC harboring truncated c1-integrons may be resistant to a greater number of antimicrobials than c1-integron-negative DEC. Structures of c1-integrons from strains in this study were compared with those from other Enterobacteriaceae (Table 5) [24–32]. Dominant resistant genes *aadA* (conferring resistance to aminoglycoside) and/or *dfrA* (conferring resistance to trimethoprim) within intact/truncated c1-integrons from strains examined in this study are also the major genes in other c1-integrons of other Enterobacteriaceae strains (Table 5). Only “*dfrA17*,” “*dfrA17*-ORF,” or “*dfrA1*-ORF” were unique gene cassettes from strains in the present study. In contrast, seven of the ten sequence patterns of cassette-borne antimicrobial and related genes in intact/truncated c1-integrons from strains studied herein have also been identified in other strains from other countries (Table 5), suggesting a worldwide circulation of the c1-integrons among Enterobacteriaceae. The cassette-borne genes identified in the present study suggest that gene cassettes within intact/truncated c1-integrons play a limited role in determining the antimicrobial resistance of Enterobacteriaceae. This indicates that the majority of antimicrobial resistance genes, except for *aadA* and *dfrA*, in DEC isolates are not cassette-borne and are located outside intact/truncated c1-integrons. Intact/truncated c1-integrons are generally associated with mobile genetic elements like transposons [10, 33, 34], which are major reservoirs of antimicrobial resistance genes. Subsequently, like strains with intact c1-integrons, DEC strains containing truncated c1-integrons might be resistant to a greater number of antimicrobials than strains without c1-integrons, as observed in the present study.

The high rates of resistance genes in EAEC isolates may be attributed to the presence of intact/truncated c1-integrons and may be promoted in animal production environments. The results of the present study align with those of previous reports showing that EAEC strains display higher rates of resistance to several antibiotics compared with other DEC pathotypes [4, 5]. In addition, significantly higher resistance rates were observed among c1-integron-positive EAEC compared with the other three DEC pathotypes (Tables 3 and 4). Moreover, our results showed that the antimicrobial resistance patterns of intact/truncated c1-integron-positive EAEC isolates were similar to those of Japanese *E. coli* isolates originating from livestock, particularly broiler chickens, although previous studies have reported that EAEC isolates from humans are characteristically divergent from those from animals [35–37]. Therefore, the high prevalence of intact/truncated c1-integrons incorporating resistance genes among EAEC in the current study suggests that these isolates may be derived from meat or meat products.

## 5. Conclusion

Regardless of whether the integrons were intact or truncated, c1-integron-positive DEC isolates examined in the current study were more frequently resistant to antibiotics than integron-negative isolates even through intact/truncated c1-integrons may only play a limited role in conferring antimicrobial resistance among DEC isolates. Thus, truncated c1-integrons may also be involved in the acquisition of antimicrobial resistance genes by DEC, particularly EAEC. Continuous surveillance is therefore required to better monitor cassette-borne resistance genes in DEC in clinical and related fields.

## Figures and Tables

**Table 1:** Diarrheagenic *Escherichia coli* strains used in this study (*n* = 162).

Pathogenic categories	No. of strains	Origin	O-serogroup	Isolation year
EAEC^*∗*^	40	Symptomatic patient (*n* = 17)	86 (*n* = 1), 111 (*n* = 1), 125 (*n* = 1), 126 (*n* = 1), 127 (*n* = 2), 153 (*n* = 1), OUT (*n* = 10)	2012–2014
		Asymptomatic carrier (*n* = 23)	44 (*n* = 1), 55 (*n* = 1), 86 (*n* = 1), 126 (*n* = 2), OUT (*n* = 18)	2012–2014
EPEC	37	Symptomatic patient (*n* = 12)	55 (*n* = 1), 114 (*n* = 1), 164 (*n* = 1), OUT (*n* = 9)	2012–2014
		Asymptomatic carrier (*n* = 25)	15 (*n* = 1), 63 (*n* = 1), 124 (*n* = 2), 125 (*n* = 1), 145 (*n* = 1), 167 (*n* = 1), OUT (*n* = 18)	2013-2014
STEC	83	Symptomatic patient (*n* = 68)	26 (*n* = 3), 103 (*n* = 3), 111 (*n* = 4), 145 (*n* = 2), 157 (*n* = 54), 165 (*n* = 1), 186 (*n* = 1)	2012–2014
		Asymptomatic carrier (*n* = 15)	26 (*n* = 3), 157 (*n* = 12)	2012–2014
ETEC	2	Asymptomatic carrier (*n* = 2)	148 (*n* = 1), 169 (*n* = 1)	2013-2014

^*∗*^EAEC: enteroaggregative *E. coli*; EPEC: enteropathogenic *E. coli*; STEC: Shiga toxin-producing *E. coli*; ETEC: enterotoxigenic *E. coli*; OUT: O-serogroup untypeable.

**Table 2:** Primers used in this study.

Target gene	Primer direction	Nucleotide sequence (5′–3′)	Amplicon size (bp)	Reference number
*intI*1	F	CAGTGGACATAAGCCTGTTC	160	15
	R	CCCGAGGCATAGACTGTA		
*sul*1	F	CGGCGTGGGCTACCTGAACG	433	15
	R	GCCGATCGCGTGAAGTTCCG		
*qacEΔ*1	F	ATCGCAATAGTTGGCGAAGT	250	15
	R	GAAGCTTTTGCCCATGAAGC		
Class 1 gene cassette	F	GGCATCCAAGCAGCAAGC	Variable	15
	R	AAGCAGACTTGACCTGAT		
*aggR*	F	GTATACACAAAAGAAGGAAGC	254	10
	R	ACAGAATCGTCAGCATCAGC		
*eae*	F	GCTTAGTGCTGGTTTAGGAT	591	10
	R	CTCTGCAGATTAACCTCTGC		

**Table 3:** Number of antibiotics to which the strains with/without integrons showed resistance.

Presence of integrons	Intact/truncated integron and pathotype	No. of strains	Number (%) of antibiotics to which each strain showed resistance^†^						
			None	One	Two	Three	Four	Five	Six
Strains with integrons	Intact integron								
	EAEC^*∗*^	11	0	1	0	0	5	3	2
	EPEC^*∗*^	2	0	0	0	0	1	0	1
	STEC^*∗*^	2	0	1	0	0	0	0	1
	ETEC^*∗*^	0	0	0	0	0	0	0	0
	Subtotal	15	1 (7%)	2 (13%)	0	0	6 (40%)	3 (20%)	3 (20%)
	Truncated integron								
	EAEC	9	0	0	0	1	2	5	1
	EPEC	3	0	0	0	1	1	1	0
	STEC	0	0	0	0	0	0	0	0
	ETEC	0	0	0	0	0	0	0	0
	Subtotal	12	0	0	0	2 (16.7)	3 (25.0)	6 (50.0)	1 (8.3)
Strains without integrons	EAEC	20	6	6	5	1	1	1	0
	EPEC	32	25	2	2	1	1	0	1
	STEC	81	66	6	7	1	1	0	0
	ETEC	2	0	1	0	0	0	1	0
	Subtotal	135	97 (71.6)	15 (11.1)	14 (10.4)	3 (2.2)	3 (2.2)	2 (1.5)	1 (0.7)

Total		162	98 (60.5)	17 (10.5)	14 (8.6)	5 (3.1)	12 (7.4)	11 (6.8)	5 (3.1)

^†^A total of 14 antimicrobials were tested (ampicillin, sulfamethoxazole-trimethoprim, tetracycline, nalidixic acid, streptomycin, chloramphenicol, gentamicin, cefotaxime, norfloxacin, kanamycin, ciprofloxacin, fosfomycin, imipenem, and meropenem).

**Table 4:** Number (%) of antibiotic-resistant diarrheagenic *Escherichia coli* isolates in Japan, with/without class 1 integrons.

Antibiotics	EAEC^*∗*^			EPEC^*∗*^			STEC^*∗*^			ETEC^*∗*^		Total	
	Integron + (*n* = 20)	Integron − (*n* = 20)	Total (*n* = 40)	Integron + (*n* = 5)	Integron − (*n* = 32)	Total (*n* = 37)	Integron + (*n* = 2)	Integron − (*n* = 81)	Total (*n* = 83)	Integron + (*n* = 0)	Integron − (*n* = 2)	Integron + (*n* = 27)	Integron − (*n* = 135)
Ampicillin	20 (100)^‡^	13 (65.0)	33 (82.5)	3 (60.0)^‡^	4 (12.5)	7 (18.9)	1 (50.0)	8 (9.9)	9 (10.8)	0	1 (50.0)	24 (88.9)^‡^	26 (19.3)
Sulfamethoxazole-trimethoprim	18 (90.0)^‡^	3 (15.0)	21 (52.5)	3 (60.0)^‡^	1 (3.1)	4 (10.8)	1 (50.0)^‡^	1 (1.2)	2 (2.4)	0	1 (50)	22 (81.5)^‡^	6 (4.4)
Tetracycline	18 (90.0)^‡^	3 (15.0)	21 (52.5)	3 (60.0)^‡^	4 (12.5)	7 (18.9)	1 (50.0)	9 (11.1)	10 (12.0)	0	1 (50.0)	22 (81.5)^‡^	17 (12.6)
Nalidixic acid	13 (65.0)^‡^	3 (15.0)	16 (40.0)	2 (40.0)	3 (9.4)	5 (13.5)	0	0	0	0	2 (100)	15 (55.6)^‡^	8 (5.9)
Streptomycin	11 (55.0)^‡^	3 (15.0)	14 (35.5)	2 (40.0)	2 (6.3)	4 (10.8)	2 (100)^‡^	6 (7.4)	8 (9.6)	0	1 (50.0)	15 (55.6)^‡^	12 (8.9)
Chloramphenicol	6 (30.0)	1 (5.0)	7 (17.5)	2 (40.0)	2 (6.3)	4 (10.8)	1 (50.0)^‡^	1 (1.2)	2 (2.4)	0	0	9 (33.3)^‡^	4 (3.0)
Gentamicin	2 (9.52)	0	2 (5.0)	0	0	0	0	0	0	0	0	2 (7.4)^‡^	0
Cefotaxime	1 (10.0)	2 (10.0)	3 (7.5)	0	2 (6.3)	2 (5.4)	0	0	0	0	0	1 (3.7)	4 (3.0)
Norfloxacin	1 (5.0)	0	1 (2.5)	0	0	0	0	0	0	0	0	1 (3.7)	0
Kanamycin	0	0	0	1 (20.0)	1 (3.1)	2 (5.4)	1 (50.0)	2 (2.5)	3 (3.6)	0	0	2 (7.4)	3 (2.2)
Ciprofloxacin, fosfomycin, imipenem, or meropenem	0	0	0	0	0	0	0	0	0	0	0	0	0
Phenotype of multidrug resistance^†^	19 (95.0)^‡^	2 (10.0)	21 (52.5)	4 (80.0)^‡^	3 (9.4)	7 (18.9)	1 (50.0)	2 (2.5)	3 (3.6)	0	1 (50.0)	24 (88.9)^‡^	8 (5.9)
*β*-lactamase genes detected	*bla*_CTX-M-14_ (*n* = 1), *bla*_TEM-1_ (*n* = 1)	*bla*_CTX-M-14_ (*n* = 1), *bla*_CTX-M-15_ (*n* = 1)	*bla*_CTX-M-14_ (*n* = 2), *bla*_CTX-M-15_ (*n* = 1), *bla*_TEM-1_ (*n* = 1)	*bla*_OXA-1_ (*n* = 1)	*bla*_CTX-M-14_ (*n* = 1), *bla*_TEM-1_ (*n* = 1)	*bla*_CTX-M-14_ (*n* = 1), *bla*_TEM-1_ (*n* = 1) *bla*_OXA-1_ (*n* = 1)						*bla*_CTX-M-14_ (*n* = 1), *bla*_TEM-1_(*n* = 1) *bla*_OXA-1_(*n* = 1)	*bla*_CTX-M-14_ (*n* = 2), *bla*_CTX-M-15_ (*n* = 1), *bla*_TEM-1_ (*n* = 1)

^†^Defined as resistance to three or more classes of antimicrobials. ^‡^Denotes significantly higher rate of resistance to antibiotics for class 1 integron-positive isolates compared with class 1 integron-negative isolates (*p* > 0.05).

**Table 5:** Comparison between this study and other studies of gene cassettes within class 1 integron.

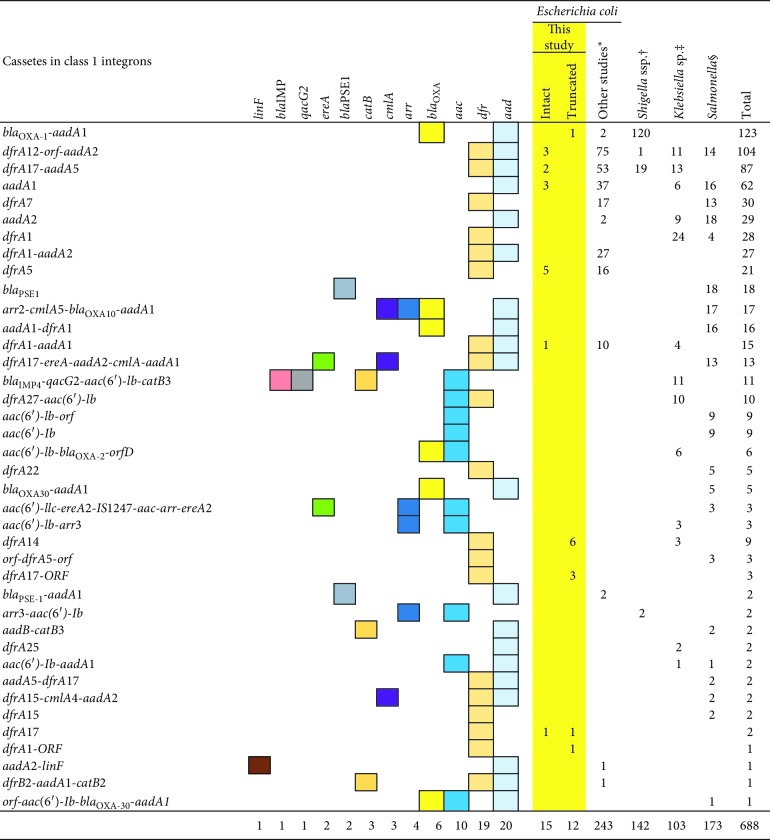

^*∗*^Kang et al. (Korea) and Heir et al. (Norway). ^†^Yang et al. (China). ^‡^Chang et al. (Taiwan), Li et al. (China) and Chowdhury et al. (Argentina, Chile, Uruguay, and Australia). ^§^Peirano et al. (Brazil), Zhang et al. (China), and Krauland et al. (United States, Canada, Argentina, Australia, Belgium, South Africa, Spain, Italy, Denmark, and Taiwan).

## Data Availability

The authors declare that raw data generated in this project are available from the corresponding authors upon reasonable request.

## Supplementary Materials

Supplementary Materials Supplementary Table 1: strains tested and accession numbers of DNA sequences deposited in the DDBJ/GenBank/EMBL database.
