# Supplementary material for: Truncated Class 1 Integron Gene Cassette Arrays Contribute to Antimicrobial Resistance of Diarrheagenic Escherichia coli
Source: Biomed Res Int. 2020 Jan 31;2020:4908189. doi: 10.1155/2020/4908189 (PMC7013361; doi:10.1155/2020/4908189)
Supplement: Supplementary Materials — Supplementary Table 1: strains tested and accession numbers of DNA sequences deposited in the DDBJ/GenBank/EMBL database. [file 4908189.f1.pdf]

Supplemental Table 1. Strains tested and accession numbers of DNA sequences deposited in the DDBJ/GenBank/EMBL database

No.	Strain	Pathogenic type	Serotype	Isolation year	*intI1*	Gene cassettes	Accession number	Run ID
1	EA1	EAEC	O44	2012	-	-	-	-
2	EA2	EAEC	O125	2012	-	-	-	-
3	EA3	EAEC	OUT	2012	+	*aadA1*	LC380541	-
4	EA4	EAEC	OUT	2012	+	*aadA1*	LC380542	-
5	EA5	EAEC	O55	2012	-	-	-	-
6	EA6	EAEC	OUT	2012	-	-	-	-
7	EA7	EAEC	OUT	2012	+	*dfrA14*	-	DRR129827
8	EA8	EAEC	OUT	2012	+	*dfrA5*	LC380543	-
9	EA9	EAEC	O153	2012	+	*dfrA1 -aadA1*	LC380544	-
10	EA10	EAEC	OUT	2012	+	*dfrA14*		DRR129828
11	EA12	EAEC	O127	2012	-	-	-	-
12	EA13	EAEC	OUT	2012	+	*dfrA17*	LC380545	-
13	EA14	EAEC	OUT	2012	+	*dfrA14*		DRR129829
14	EA15	EAEC	OUT	2013	+	*dfrA14*		DRR129830
15	EA20	EAEC	O86	2013	+	*aadA1*	LC380546	
16	EA22	EAEC	O111	2013	-	-	-	-
17	EA23	EAEC	OUT	2013	-	-	-	-
18	EA24	EAEC	O126	2013	-	-	-	-
19	EA25	EAEC	OUT	2013	+	*dfrA5*	LC380547	-
20	EA26	EAEC	OUT	2013	+	*dfrA5*	LC380548	-
21	EA28	EAEC	OUT	2013	-	-	-	-
22	EA29	EAEC	O126	2013	-	-	-	-
23	EA36	EAEC	O126	2013	-	-	-	-
24	EA37	EAEC	OUT	2013	-	-	-	-
25	EA38	EAEC	OUT	2013	-	-	-	-
26	EA39	EAEC	O86	2013	-	-	-	-
27	EA40	EAEC	OUT	2013	-	-	-	-
28	EA41	EAEC	OUT	2014	+	*dfrA14*	-	DRR129831
29	EA42	EAEC	OUT	2014	-	-	-	-
30	EA43	EAEC	OUT	2014	+	*dfrA12 -aadA2*	LC380549	-
31	EA44	EAEC	OUT	2014	+	*dfrA17*		DRR129832
32	EA45	EAEC	OUT	2014	-	-	-	-
33	EA46	EAEC	OUT	2014	-	-	-	-
34	EA50	EAEC	OUT	2014	+	*dfrA17*	-	DRR129833
35	EA51	EAEC	OUT	2014	+	*dfrA17*	-	DRR129834
36	EA60	EAEC	OUT	2014	-	-	-	-
37	EA61	EAEC	OUT	2014	+	*dfrA5*	LC380550	-
38	EA62	EAEC	OUT	2014	+	*dfrA5*	LC380551	-
39	EA63	EAEC	OUT	2014	+	*dfrA17*	-	DRR129835
40	EA64	EAEC	O127	2014	-	-	-	-
41	EP1	EPEC	OUT	2012	+	*dfrA17 -aadA5*	LC380554	-
42	EP2	EPEC	O114	2012	-	-	-	-
43	EP3	EPEC	OUT	2012	-	-	-	-
44	EP4	EPEC	OUT	2012	-	-	-	-
45	EP5	EPEC	OUT	2012	-	-	-	-
46	EP9	EPEC	O15	2013	-	-	-	-
47	EP10	EPEC	O145	2013	-	-	-	-
48	EP11	EPEC	O125	2013	-	-	-	-
49	EP13	EPEC	OUT	2013	-	-	-	-
50	EP14	EPEC	O124	2013	-	-	-	-
51	EP15	EPEC	OUT	2013	-	-	-	-
52	EP16	EPEC	O124	2013	-	-	-	-
53	EP17	EPEC	O63	2013	-	-	-	-
54	EP18	EPEC	OUT	2013	-	-	-	-
55	EP19	EPEC	OUT	2013	-	-	-	-
56	EP21	EPEC	OUT	2013	-	-	-	-
57	EP23	EPEC	OUT	2013	-	-	-	-
58	EP24	EPEC	OUT	2013	-	-	-	-
59	EP25	EPEC	OUT	2013	-	-	-	-

60	EP26	EPEC	OUT	2013	-	-	-	-
61	EP27	EPEC	OUT	2013	+	*dfrA17*-*aadA5*	LC383355	
62	EP28	EPEC	OUT	2013	-	-	-	-
63	EP29	EPEC	OUT	2013	-	-	-	-
64	EP30	EPEC	O55	2013	-	-	-	-
65	EP31	EPEC	OUT	2013	-	-	-	-
66	EP32	EPEC	O164	2013	-	-	-	-
67	EP33	EPEC	OUT	2013	-	-	-	-
68	EP34	EPEC	OUT	2013	-	-	-	-
69	EP35	EPEC	OUT	2014	+	*bla* $_{OXA}$-*aadA1*	-	DRR129836
70	EP36	EPEC	OUT	2014	-	-	-	-
71	EP37	EPEC	OUT	2014	-	-	-	-
72	EP38	EPEC	O167	2014	-	-	-	-
73	EP39	EPEC	OUT	2014	+	*dfrA14*	-	DRR129837
74	EP40	EPEC	OUT	2014	-	-	-	-
75	EP41	EPEC	OUT	2014	-	-	-	-
76	EP42	EPEC	OUT	2014	+	*dfrA1*	-	DRR129838
77	EP43	EPEC	OUT	2014	-	-	-	-
78	EH1	STEC	O157	2012	-	-	-	-
79	EH2	STEC	O165	2012	-	-	-	-
80	EH3	STEC	O157	2012	-	-	-	-
81	EH4	STEC	O26	2012	-	-	-	-
82	EH5	STEC	O111	2012	-	-	-	-
83	EH6	STEC	O157	2012	-	-	-	-
84	EH7	STEC	O26	2012	-	-	-	-
85	EH8	STEC	O157	2012	-	-	-	-
86	EH9	STEC	O157	2012	-	-	-	-
87	EH10	STEC	O157	2012	-	-	-	-
88	EH11	STEC	O157	2012	-	-	-	-
89	EH12	STEC	O157	2012	-	-	-	-
90	EH13	STEC	O157	2012	-	-	-	-
91	EH14	STEC	O157	2012	-	-	-	-
92	EH15	STEC	O157	2012	-	-	-	-
93	EH16	STEC	O157	2012	-	-	-	-
94	EH17	STEC	O157	2012	-	-	-	-
95	EH18	STEC	O157	2012	-	-	-	-
96	EH19	STEC	O157	2012	-	-	-	-
97	EH20	STEC	O157	2013	-	-	-	-
98	EH21	STEC	O157	2013	-	-	-	-
99	EH22	STEC	O157	2013	-	-	-	-
100	EH23	STEC	O103	2013	-	-	-	-
101	EH24	STEC	O157	2013	-	-	-	-
102	EH25	STEC	O111	2013	-	-	-	-
103	EH26	STEC	O157	2013	-	-	-	-
104	EH27	STEC	O157	2013	-	-	-	-
105	EH28	STEC	O111	2013	+	*dfrA12*-*aadA2*	LC380552	-
106	EH29	STEC	O157	2013	-	-	-	-
107	EH30	STEC	O157	2013	-	-	-	-
108	EH31	STEC	O157	2013	-	-	-	-
109	EH32	STEC	O157	2013	-	-	-	-
110	EH33	STEC	O157	2013	-	-	-	-
111	EH34	STEC	O145	2013	-	-	-	-
112	EH35	STEC	O157	2013	-	-	-	-
113	EH36	STEC	O157	2013	-	-	-	-
114	EH37	STEC	O157	2013	-	-	-	-
115	EH38	STEC	O157	2013	-	-	-	-
116	EH39	STEC	O157	2013	-	-	-	-
117	EH40	STEC	O145	2013	+	*dfrA12*-*aadA2*	LC380553	-
118	EH41	STEC	O157	2013	-	-	-	-
119	EH42	STEC	O157	2013	-	-	-	-
120	EH43	STEC	O186	2014	-	-	-	-
121	EH44	STEC	O157	2014	-	-	-	-
122	EH45	STEC	O157	2014	-	-	-	-

123	EH46	STEC	O26	2014	-	-	-	-
124	EH47	STEC	O157	2014	-	-	-	-
125	EH48	STEC	O157	2014	-	-	-	-
126	EH49	STEC	O157	2014	-	-	-	-
127	EH50	STEC	O157	2014	-	-	-	-
128	EH51	STEC	O157	2014	-	-	-	-
129	EH52	STEC	O157	2014	-	-	-	-
130	EH53	STEC	O157	2014	-	-	-	-
131	EH54	STEC	O157	2014	-	-	-	-
132	EH55	STEC	O157	2014	-	-	-	-
133	EH56	STEC	O157	2014	-	-	-	-
134	EH57	STEC	O157	2014	-	-	-	-
135	EH58	STEC	O157	2014	-	-	-	-
136	EH59	STEC	O26	2014	-	-	-	-
137	EH60	STEC	O157	2014	-	-	-	-
138	EH61	STEC	O26	2014	-	-	-	-
139	EH62	STEC	O157	2014	-	-	-	-
140	EH63	STEC	O103	2014	-	-	-	-
141	EH64	STEC	O157	2014	-	-	-	-
142	EH65	STEC	O157	2014	-	-	-	-
143	EH66	STEC	O157	2014	-	-	-	-
144	EH67	STEC	O111	2014	-	-	-	-
145	EH68	STEC	O157	2014	-	-	-	-
146	EH69	STEC	O26	2014	-	-	-	-
147	EH70	STEC	O157	2014	-	-	-	-
148	EH71	STEC	O157	2014	-	-	-	-
149	EH72	STEC	O157	2014	-	-	-	-
150	EH73	STEC	O157	2014	-	-	-	-
151	EH74	STEC	O157	2014	-	-	-	-
152	EH75	STEC	O157	2014	-	-	-	-
153	EH76	STEC	O157	2014	-	-	-	-
154	EH77	STEC	O157	2014	-	-	-	-
155	EH78	STEC	O157	2014	-	-	-	-
156	EH79	STEC	O157	2014	-	-	-	-
157	EH80	STEC	O157	2014	-	-	-	-
158	EH81	STEC	O157	2014	-	-	-	-
159	EH82	STEC	O103	2014	-	-	-	-
160	EH83	STEC	O157	2014	-	-	-	-
161	ET1	ETEC	O148	2013	-	-	-	-
162	ET2	ETEC	O169	2014	-	-	-	-
